# Structural Characterization and Antitumor Activity of Polysaccharides from *Kaempferia galanga* L.

**DOI:** 10.1155/2018/9579262

**Published:** 2018-12-30

**Authors:** Xu Yang, Haiyu Ji, Yingying Feng, Juan Yu, Anjun Liu

**Affiliations:** ^1^Key Laboratory of Food Nutrition and Safety, Ministry of Education, School of Food Engineering and Biotechnology, Tianjin University of Science and Technology, Tianjin 300457, China; ^2^QingYunTang Biotech (Beijing) Co. Ltd., Beijing 100176, China

## Abstract

The water-soluble polysaccharides from *Kaempferia galanga* L. (KGPs) were extracted and purified, and their structural characteristics and antitumor activity were further investigated. The UV spectrum, high-performance gel permeation chromatography (HPGPC), Fourier-transform infrared spectroscopy (FTIR), and ion chromatography (IC) were employed to evaluate the structural characteristics, and H22 tumor-bearing mice model was established to demonstrate the antitumor activity. Physicochemical analysis and UV spectrum results showed that the proportions of total sugar, protein, and uronic acid in KGPs were 85.23%, 0.54%, and 24.17%, respectively. HPGPC, FTIR, and IC indicated that KGPs were acidic polysaccharides with skeletal modes of pyranose rings and mainly composed of arabinose and galactose with the average molecular weight of 8.5 × 10^5^ Da. The *in vivo* antitumor experiments showed that KGPs could effectively protect the thymus and spleen of tumor-bearing mice from solid tumors and enhance the immunoregulatory ability of CD4^+^ T cells, the cytotoxic effects of CD8^+^ T cells and NK cells, and finally resulting in the inhibitory effects on H22 solid tumors. This study provided a theoretical foundation for the practical application of KGPs in food and medical industries.

## 1. Introduction

*Kaempferia galanga* L. is an acaulescent perennial plant growing in China [1], and the rhizome has been widely used as indigenous medicine due to the various bioactive compounds, including essential oils [2] and other extracts by methanol [3], hexane [4], and ethanol [5, 6]. As reported, these bioactive substances have exhibited antioxidant, sedative, antitumor, anti-inflammatory, and antimicrobial activities [7], which would contribute to the application on curing many diseases. The methanol extract of *Kaempferia galanga* L. rhizome significantly reduced viable Ehrlich ascites carcinoma (EAC) cells and weight gain, increased life span, and restored all hematological parameters, such as RBC, WBC, and hemoglobin of EAC-bearing mice towards normal level [8]. In addition, the ethanolic extract of *Kaempferia galanga* L. rhizome, ethyl-p-methoxycinnamate, exhibited promising anticholangiocarcinoma activity in CL6-xenografed nude mice as determined by significant inhibitory activity on tumor growth and lung metastasis, as well as prolongation of survival time [5], which could inhibit the proliferation of human hepatocellular liver carcinoma cells in a dose-dependent manner by inducing cells to enter into apoptosis [6]. However, there are few researches concerning the polysaccharides from the rhizome of *Kaempferia galanga* L. and their *in vivo* antitumor activities.

Polysaccharides commonly exist in various animals, plants, fungus, and algae as polymeric carbohydrate molecule and have exhibited anticoagulant, antioxidant, antitumor, and immunoregulatory activities [9–11] with relatively low toxicity [12]. As is known to us, the properties and bioactivities of polysaccharides were directly associated with their chemical characteristics [13], which could be influenced by extraction techniques [14, 15]. Hot water extraction has been commonly employed to extract crude polysaccharides due to the simple process and low cost [16, 17].

Hepatocellular carcinoma (HCC) is the third leading cause of cancer-related mortality worldwide as one of the most common malignant tumors, which has been a major public health problem [18, 19]. Modern therapeutic methods of treating HCC patients are chemotherapy [20], but the curative effects are poor due to the relapse of disease and resistance to chemotherapeutics [21]. Therefore, many natural products with stronger antitumor activity and lower toxicity were studied in recent years.

Previous studies mainly focused on the extracts from *Kaempferia galanga* L. by organic reagents and their bioactivities, while the relevant researches about the polysaccharides have not been reported yet. In the present study, the water-soluble polysaccharides from *Kaempferia galanga* L. (KGPs) were extracted with hot water, and their structural characteristics and antitumor effects were further evaluated.

## 2. Materials and Methods

### 2.1. Materials and Reagents

The rhizomes (*Kaempferia galanga* L.) were collected from Zhanjiang city (Guangdong, China) and shattered after dried to constant weight. H22 hepatoma cells were obtained from the Shanghai Institute of Biological Sciences at the Chinese Academy of Sciences (Shanghai, China). Bovine serum albumin (BSA), glucuronic acid, trifluoroacetic acid (TFA), standard monosaccharides, dimethyl sulfoxide (DMSO), 3-(4,5-dimethylthiazol-2-yl)-2,5-diphenyltetrazolium bromide (MTT), and 5-fluorouracil (5-FU) were purchased from Solarbio (Beijing, China). Fetal bovine serum (FBS) was obtained from Hangzhou Sijiqing Co. (Hangzhou, China). All other chemicals and agents were of analytical grade.

### 2.2. Preparation of Polysaccharides

The powder of rhizomes (100 g) was soaked in distilled water (3 L) for 4 h at 80°C two times. Then, the insoluble components were removed by centrifugation, supernatant was collected, and 4 volumes of ethanol were added for precipitating. The precipitations were kept and redissolved in deionized water; Sevag method (1-butanol and chloroform (1 : 4, v/v) for 5 times) was used to remove proteins [22]. Finally, the mixture was purified by a Sephadex G-200 column (1.6 cm × 35 cm) eluted with distilled water at 1 mL/min and lyophilized to obtain the *Kaempferia galanga* L. polysaccharides (KGPs, ~2.56 ± 0.12 g per 100 g dry weight of rhizomes) for further research.

### 2.3. Chemical Composition and Structural Analysis

Total sugar content was determined by phenol/H_2_SO_4_ assay and glucose was used as standard [23]. The content of uronic acid was detected according to the carbazole-sulfuric method using glucuronic acid as the standard [24]. The protein content was evaluated by the Coomassie brilliant blue method with bovine serum albumin as the standard [25]. The samples (5 mg) were dissolved in deionized water (1 mg/mL) and scanned from 200 to 800 nm with a spectrum-2102 UV spectrophotometer.

HPGPC (Agilent-1200) and FTIR (Bruker VECTOR-22, Karlsruhe, Germany) were employed to determine the average molecular weight and bonded states of the polysaccharides following the previous methods [26]. The standard curve of average molecular weight was established using the T-series dextrans (T-10, T-40, T-70, T-500, and T-2000).

The monosaccharide constituents and proportions were determined by IC, a Dionex ICS2500 chromatographic system (CA, USA) with a Dionex pulsed amperometric detector with an Au electrode and an efficient anion exchange column of Dionex Carbopac PA20 column (150 mm × 3 mm) [27]. The polysaccharide sample (5 mg) was added into 1 mL of 2 M trifluoroacetic acid (TFA) and hydrolyzed at 110°C for 4 h in a sealed tube. Then TFA was absolutely removed by adding methanol and N_2_ was used as the carrier gas. The dried hydrolysate was dissolved in 1 mL of distilled water, diluted 10 times with distilled water. The solution was eluted with NaOH (6 mM) and NaAC (100 mM) solutions at a flow rate of 0.45 mL/min. D-fucose, D-rhamnose, L-arabinose, D-galactose, D-glucose, L-xylose, D-mannose, D-glucuronic acid, and D-galacturonic acid were used as the standards for calibration and quantification.

### 2.4. Antitumor Animal Experiment

#### 2.4.1. Design of Animal Model

Female KM mice of SPF-level (6-8 weeks old, 18-22 g) were purchased from the Center of Experimental Animals of Academy of Military Science (Beijing, China). They were raised under pathogen-free conditions with the 22 ± 2°C temperature, 50 ± 10% humidity, and 12 h light/12 h dark cycle. All mice were permitted free access to tap water and fed with standard pellet diet and to acclimate to new environments for 1 week before the experiment. All animal experimental procedures were conducted in accordance with the principles of Laboratory Animal Care and approved by the Local Ethics Committee for Animal Care and Use at Tianjin University of Science and Technology.

60 healthy female mice were randomly separated into six groups with 10 mice each group: blank group, model group, KGPs groups (100, 200, and 300 mg/kg KGPs) and positive group (5-Fu, 30 mg/kg, which has been in use against cancer for about 40 years [28]). The blank and model group mice were orally administrated with saline, while KGP groups were gavaged with different concentrations of KGPs, the positive group was intraperitoneally infused with 5-Fu. On day 15, 0.2 mL murine H22 hepatoma cells (1 × 10^7^ cells/mL) were inoculated in the subcutaneous right forelimb armpit of mice except the blank group. Then all mice were sequentially treated for another 15 days. Finally, they were all sacrificed by cervical dislocation and the tumors, spleens, and thymuses were weighed immediately. Meanwhile, the blood, spleen, and thymus samples were prepared and analyzed.

The immune organ indexes were expressed as the organ weight relative to body weight, and tumor inhibitory ratio was calculated by the following formula: Inhibitory rate (%) = [(*W*_1_ − *W*_2_)/*W*_1_] × 100 [29], *W*_1_ represents the average tumor weight of the model group and *W*_2_ represents the average tumor weight of the treated group.

#### 2.4.2. Blood Routine Parameter Examination

Anticoagulant (EDTA-K_2_) was added into the blood sample to prevent clot forming, and all samples were analyzed on the XFA-6130 automatic blood analyzer.

#### 2.4.3. Splenic Lymphocyte Proliferation and NK Cell Activity

Splenic lymphocyte proliferative activity (T cells and B cells stimulated with ConA and LPS, respectively) and NK cell activity (killing activity on H22 hepatoma cells) were performed as described previously [30].

#### 2.4.4. Assessment of Lymphocyte Subsets in Peripheral Blood

The proportions of lymphocyte subsets in the peripheral blood were detected using FCM assay. The bloods were obtained and stained with monoclonal antibodies (mAb) against CD3-FITC, CD19-PE, CD4-PE, and CD8-FITC for 30 min on ice in the dark. Then the erythrocytes and uncombined antibodies were removed by red blood cell lysis buffer and phosphate buffer washing, and finally, a flow cytometry (BD FACSCalibur, Becton Dickinson, Franklin Lakes, NJ, USA) performed with CellQuest Pro software (version 5.1, Becton Dickinson, Franklin Lakes, NJ, USA) was employed to measure the fluorescently labeled lymphocyte subsets (counting 10,000 events).

#### 2.4.5. Histological Observations of the Thymus and Spleen Organ

The thymus and spleen organs of the mice were fixed in 10% neutral formaldehyde solution and dehydrated in gradient ethanol solution. After embedding in paraffin, 4 *μ*m sections were obtained and stained with hematoxylin and eosin (H&E) for microscopic examination.

### 2.5. Statistical Analysis

All values were presented as the mean ± standard deviation (S.D.) of three independent experiments performed in triplicate and statistically analyzed using SPSS for windows, version 19.0 (SPSS Inc., Chicago, IL, USA). The significance of difference was analyzed by one-way analysis of variance (ANOVA) followed by Duncan's test and *p* < 0.05 of the data was considered to be significant.

## 3. Results

### 3.1. Chemical Composition of KGPs

**Figure 1:**
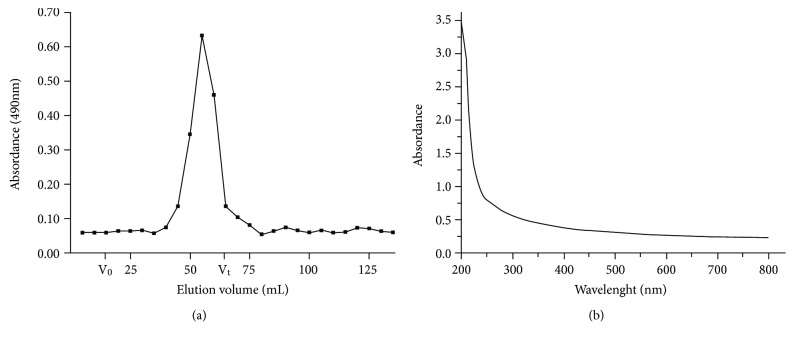
The elution curve via Sephadex G-200 column (a) and UV spectrum in the range of 200-800 nm of KGPs (b). *V*_0_ and *V*_*t*_ are the void volume 15.1 mL and total column volume 62.2 mL, respectively.

The proportions of total sugar, protein, and uronic acid in KGPs were 85.23%, 0.54%, and 24.17%, respectively, which indicated that KGPs were acidic polysaccharides with little amount of protein.  Figure 1(a) shows the elution curve via Sephadex G-200 column and UV spectrum in the range of 200-800 nm of KGPs; the results indicated that KGPs were purified well with little proteins due to no absorptions at 260 nm and 280 nm [31].

### 3.2. Molecular Weight Distribution and FTIR Spectra Analysis

**Figure 2:**
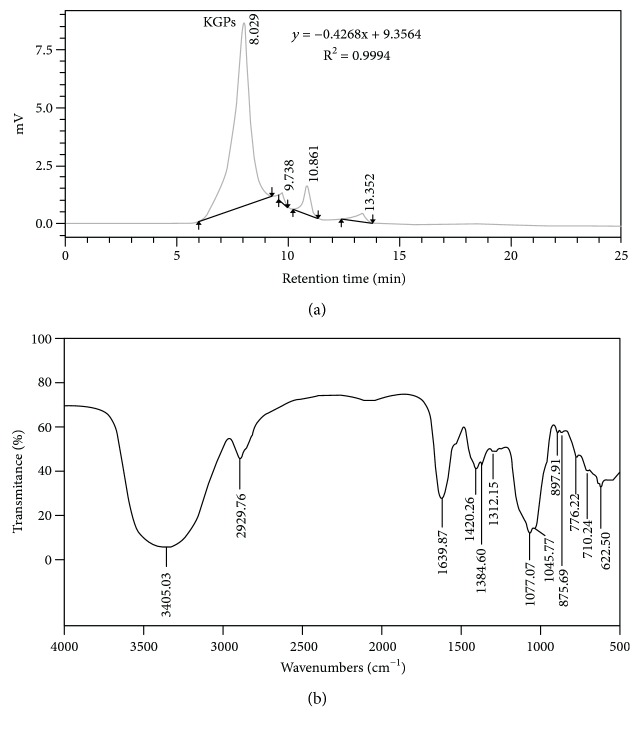
The results of HPGPC (a) and FTIR spectra (b) in the range of 500-4000 cm^−1^ of KGPs.

The average molecular weight of KGPs was determined by HPGPC method, and the result is shown in Figure 2(a). The HPGPC profiles of KGPs presented one main peak at 8.029 min, which occupied 93.15% of the total area. The molecular weight of KGPs was 8.5 × 10^5^ Da according to the standard curve performed by glucans.  Figure 2(b) exhibited the major functional groups and the chemical bounds of the polysaccharides. As shown, the broad absorption at 3405.03 cm^−1^ corresponded to the O-H stretching vibration [32], and the strong band at 2929.76 cm^−1^ was ascribed to stretching vibrations of C-H [33]. Two strong bands at 1639.87 and 1420.26 cm^−1^ were assigned to the absorbance of the deprotonated carboxylic group (COO-) [34]. These characteristics indicated that KGPs were typical acidic polysaccharides, which agreed with the content of uronic acid (24.17%). The absorptions at 500-900 cm^−1^ and 1077.07 cm^−1^ were assigned to the skeletal modes of pyranose rings [35, 36].

### 3.3. Monosaccharide Analysis of KGPs

**Figure 3:**
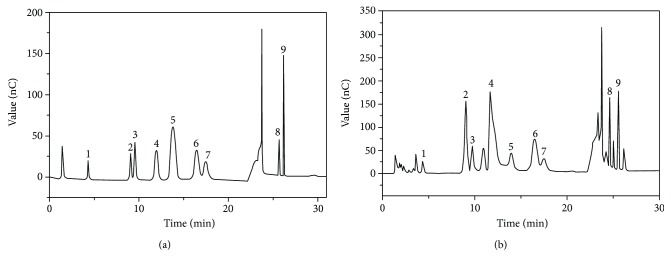
The IC results of standard monosaccharides (a) and KGPs (b). 1: fucose; 2: arabinose; 3: xylose; 4: galactose; 5: glucose; 6: rhamnose; 7: mannose; 8: glucuronic acid; 9: galacturonic acid.

IC was used to evaluate the monosaccharide composition of KGPs, and the results are shown in Figure 3. As shown, KGPs were composed of fucose, arabinose, xylose, galactose, glucose, rhamnose, mannose, glucuronic acid, and galacturonic acid in a molar ratio of 0.37 : 3.12 : 1.23 : 6.39 : 1.36 : 3.09 : 1.00 : 0.91 : 1.27, strongly suggesting that KGPs were heterogeneous acidic polysaccharides and mainly composed of arabinose and galactose.

### 3.4. Antitumor Animal Experiment

#### 3.4.1. Organ Indexes and Inhibitory Rate

**Figure 4:**
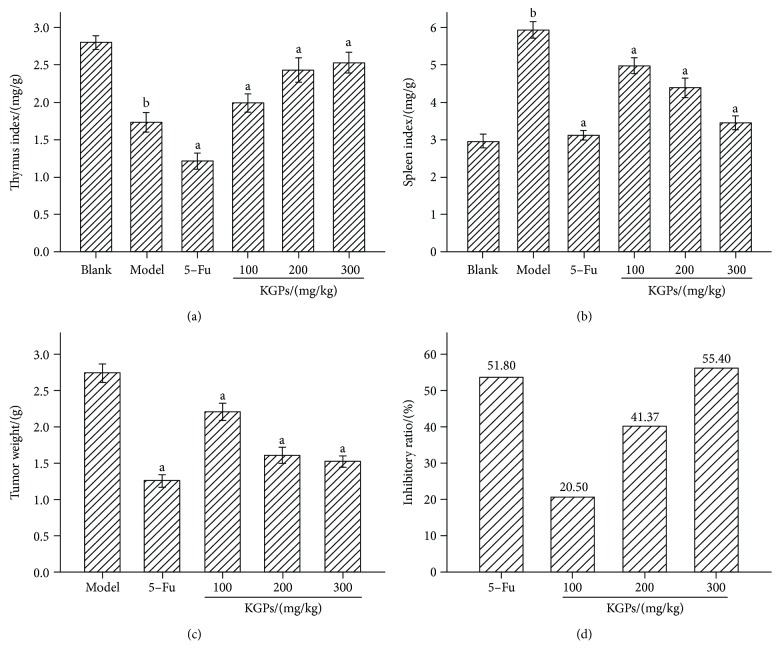
The organ indexes and inhibitory rate of H22 tumor-bearing mice. (a) Thymus index. (b) Spleen index. (c) Tumor weight. (d) Inhibitory rate. ^a^*p* < 0.05 compared to the model group; ^b^*p* < 0.05 compared to the blank group.

**Figure 5:**
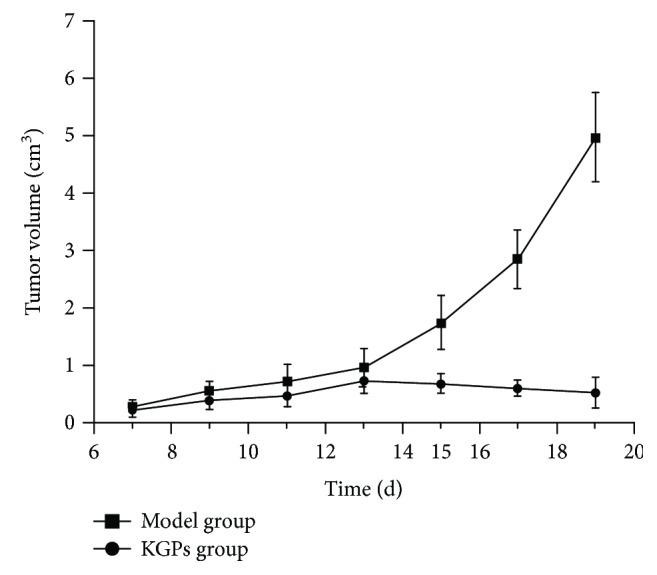
The changes of tumor volume of H22 tumor-bearing mice.

The antitumor activity of KGPs *in vivo* was further investigated on H22 tumor-bearing mice. The antitumor activities of 5-Fu and different dosages of KGPs are summarized in Figure 4. The thymus indexes of the model group were significantly reduced compared to those of the blank group (*p* < 0.05), while their spleen indexes were remarkably increased (*p* < 0.05), indicating that the proliferation of H22 cells *in vivo* could destroy immune organs. KGPs could effectively protect the thymus and spleen from solid tumors in the host, while 5-Fu exhibited strong inhibitory effects on both tumors and immune organs. The inhibition rates of tumor growth of 5-Fu and KGP treatments (100, 200, and 300 mg/kg) were 51.80%, 20.50%, 41.37%, and 55.40%, respectively. As shown in Figure 5, the H22 solid tumor volume of the model group trended to increase continually with the time varied from 7 d to 19 d, while that of the KGP group raised slowly as the time changed from 7 d to 13 d and reached to the maximum at 13 d, then decrease gradually. The H22 solid tumor volume became significant differences (*p* < 0.05) between the model group and the KGP group at 15 d. The results showed that KGPs could significantly inhibit the proliferation of tumors and protect the immune system of tumor-bearing mice, dose-dependently.

#### 3.4.2. Results of Blood Routine Examination

**Figure 6:**
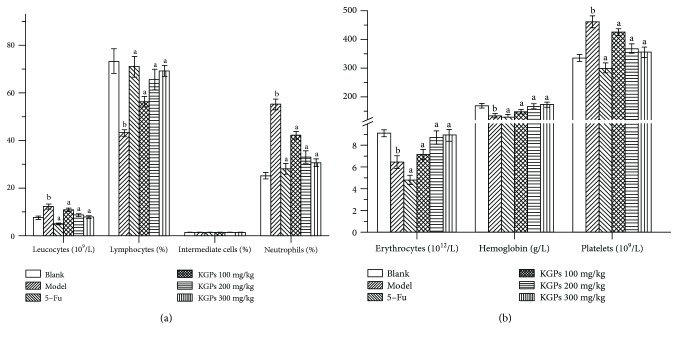
The blood routine examination of H22 tumor bearing-mice. ^a^*p* < 0.05 compared to the model group; ^b^*p* < 0.05 compared to the blank group.

The blood routine examination of tumor-bearing mice was detected in the present study, and the results are shown in Figure 6. As shown, the percentage of lymphocytes, erythrocyte counts, and content of hemoglobin in the peripheral blood of the model group were significantly decreased compared to those of the blank group (*p* < 0.05), whereas the leucocyte counts, neutrophil percentage, and platelet counts remarkably ascended (*p* < 0.05), indicating that the tumor-bearing mice in the model group had signs of inflammation and anemia. These indicators in the 5-Fu group were all lower than those in the model group, suggesting its severely toxic side effects. However, KGPs could remarkably alleviate the symptoms and improve the percentage of lymphocytes in H22 tumor-bearing mice with a dose-dependent manner.

#### 3.4.3. Spleen Lymphocyte Proliferation and NK Cell Killing Activity

**Figure 7:**
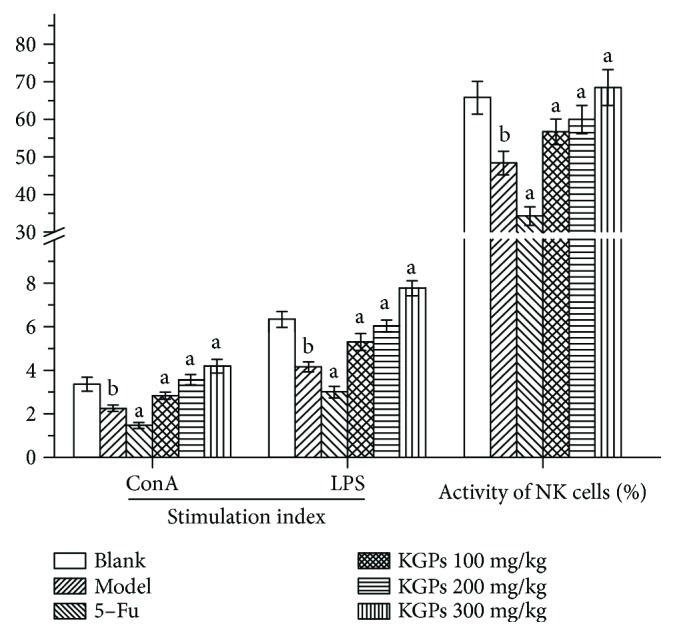
Effects of KGPs on the spleen lymphocyte proliferation and NK cell activity. ^a^*p* < 0.05 compared to the model group; ^b^*p* < 0.05 compared to the blank group.

The proliferation ability of splenic lymphocytes induced by ConA and LPS and the NK cell killing activity in H22 tumor-bearing mice were determined by MTT assay.  Figure 7 shows that the stimulation indexes (ConA and LPS) and NK cell activity of the model group were significantly reduced compared with those of the blank group (*p* < 0.05); these indicators in the 5-Fu group were even lower than those in the model group. In contrast, KGPs could dramatically improve the proliferation ability of T cells and B cells and the killing activity of NK cells compared with the model group (*p* < 0.05) in a dose-dependent manner, suggesting that KGPs could enhance the antitumor immunity of tumor-bearing host.

#### 3.4.4. Distribution of Lymphocyte Subsets

**Figure 8:**
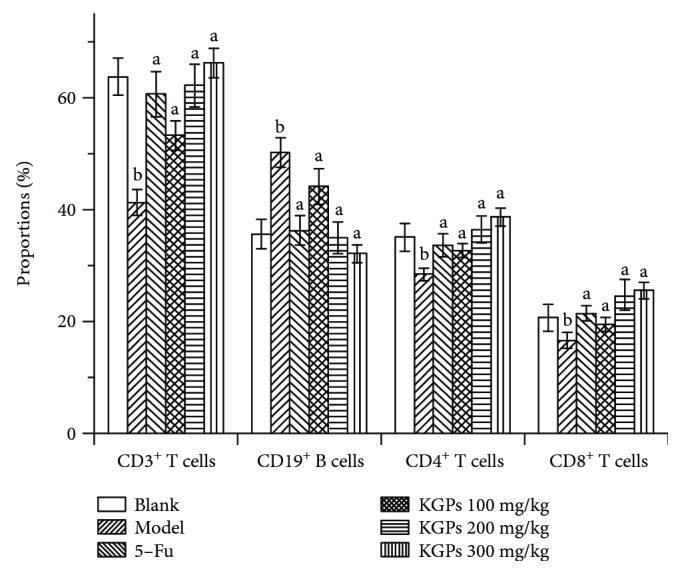
Distribution of lymphocyte subsets in the peripheral blood of H22 tumor-bearing mice. ^a^*p* < 0.05 compared to the model group; ^b^*p* < 0.05 compared to the blank group.

The proportions of lymphocyte subsets in the peripheral bloods of H22 tumor-bearing mice were researched and the results are shown in Figure 8. The percentages of T cells (CD3^+^, CD4^+^, and CD8^+^) in the model group were markedly decreased (*p* < 0.05) in relation to the blank group, which would contribute to the higher level of B cells (CD19^+^). Mice in 5-Fu showed a relatively balanced distribution of lymphocyte subsets, suggesting that 5-Fu had similar toxicity on both T cells and B cells considering the decrease in lymphocyte counts (Figure 6). The proportions of T cells in the KGP group were significantly increased (*p* < 0.05) compared to those in the model group, dose-dependently, especially the proportion of CD8^+^ T cells.

#### 3.4.5. H&E Staining of the Thymus and Spleen

**Figure 9:**
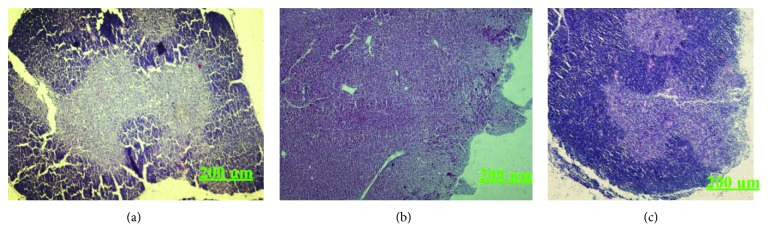
H&E staining of the thymus of H22 tumor-bearing mice. (a) Blank group. (b) Model group. (c) KGP group.

**Figure 10:**
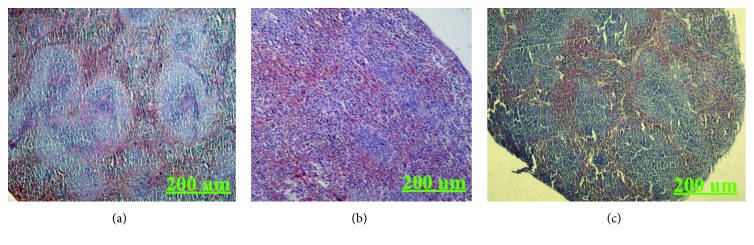
H&E staining of the spleen of H22 tumor-bearing mice. (a) Blank group. (b) Model group. (c) KGP group.

The histological observations of the thymus of H22 tumor-bearing mice are shown in Figure 9. Compared to the blank group, the thymus lobules of the model group were differentiated indistinctly. There was no clear boundary between the medullae. While the thymus lobules of KGP group were split obviously, the cortical area was increased conspicuously and the structure of the thymus was recovered gradually. As shown in Figure 10, the boundary between the red and white medullae of the KGP group was evident, and there was no significant difference with that of the blank group. It was indicated that KGPs could protect the thymus and spleen organs of H22 tumor-bearing mice.

## 4. Discussion

Radical surgery including chemotherapy has traditionally been the medical treatments for liver cancer. However, it would make the patients suffer high costs and recurrence [37]. Therefore, more effective drugs need to be investigated. Multiple polysaccharides have exhibited strong antitumor activity *in vivo* with relatively low toxicity [38, 39]. As reported, the antitumor activity of polysaccharides was closely related to their molecular weight, functional groups, monosaccharide composition, and so on [40]. Previous studies have proved that polysaccharides with the pyranose form and uronic acid would exhibit strong antitumor and immunomodulatory activities on tumor-bearing mice [26, 41, 42]. In the present study, KGPs were acidic polysaccharides (uronic acid of 24.17%) with skeletal modes of pyranose rings and mainly composed of arabinose and galactose with the average molecular weight of 8.5 × 10^5^ Da, which were consistent with characteristics of bioactive polysaccharides reported.

The thymus is a primary lymphoid organ that takes charge of differentiation and maturation of immunocompetent T lymphocytes [43], and T lymphocytes are the main protagonists in orchestrating the antitumor response including CD8^+^ T cells and CD4^+^ T cells [44]. The spleen, as a peripheral lymphoid organ, plays a central role in host defense. The damage of the spleen is related to immunodeficiency, resulting in overwhelming infections and insufficient hematopoiesis [45]. In this study, the growth of solid tumor cells *in vivo* would destroy the structure and function of the thymus and spleen, leading to the immunosuppression, inflammation, and hypohemia of the host. KGP treatment could protect these immune organs from solid tumors and balance the proportions and quantities of leukocytes, thus enhance the antitumor immunity of tumor-bearing mice.

CD4^+^ T cells can modulate antitumor immune response in antitumor immunity via activating CD8^+^ T cells and NK cells; CD8^+^ T cells impart cytolytic activity on tumor cells [46]. NK cells belong to the innate lymphoid cell family and participate in damaging infected and cancerous cells [47]. Inflammation would increase the degree of CD19^+^ B cells as reported [48]. Our results showed that mice of the model group were infected due to the malignant proliferation of H22 hepatoma cells, which was consistent with the higher proportion of CD19^+^ B cells. KGPs could notably enhance the immunoregulation capability of CD4^+^ T cells and the cytotoxic effects of CD8^+^ T cells and NK cells, finally leading to the inhibitory effects on the growth of H22 solid tumors.

## 5. Conclusions

In conclusion, we extracted and purified the polysaccharides from *Kaempferia galanga L.* (KGPs) and researched the structural characteristics and antitumor activity on H22 tumor-bearing mice. Our results showed that KGPs were acidic polysaccharides (total sugar of 85.23%, uronic acid of 24.17%) with skeletal modes of pyranose rings and mainly composed of arabinose and galactose with the average molecular weight of 8.5 × 10^5^ Da. The *in vivo* antitumor test showed that KGPs could effectively protect the thymus and spleen of tumor-bearing mice from solid tumors and enhance the immunoregulatory capability of CD4^+^ T cells and the cytotoxic effects of CD8^+^ T cells and NK cells, finally leading to the inhibitory effects on H22 solid tumors. This study provided a theoretical basis for the practical application of the novel acidic polysaccharides in food and medical industries.

## Data Availability

Data of the compounds are not available from the authors.
